# Impact of homologous and heterologous boosters in neutralizing antibodies titers against SARS-CoV-2 Omicron in solid-organ transplant recipients

**DOI:** 10.3389/fimmu.2023.1135478

**Published:** 2023-03-13

**Authors:** Aracelly Gaete-Argel, Vicente Saavedra-Alarcón, Denis Sauré, Luis Alonso-Palomares, Mónica L. Acevedo, Marion Alarcón, Susan M. Bueno, Alexis M. Kalergis, Ricardo Soto-Rifo, Fernando Valiente-Echeverría, Claudia P. Cortes

**Affiliations:** ^1^ Laboratorio de Virología Molecular y Celular, Programa de Virología, Instituto de Ciencias Biomédicas, Facultad de Medicina, Universidad de Chile, Santiago, Chile; ^2^ Millennium Institute on Immunology and Immunotherapy, Santiago, Chile; ^3^ Departamento de Ingenieria Industrial, Facultad de Ciencias Físicas y Matemáticas, University of Chile and Institutos Sistemas Complejos de Ingenieria, Santiago, Chile; ^4^ Clínica Santa María, Santiago, Chile; ^5^ Millennium Institute on Immunology and Immunotherapy, Departamento de Genética Molecular y Microbiología, Facultad de Ciencias Biológicas, Pontificia Universidad Católica de Chile, Santiago, Chile; ^6^ Departamento de Endocrinología, Facultad de Medicina, Escuela de Medicina, Pontificia Universidad Católica de Chile, Santiago, Chile; ^7^ Departamento de Medicina Interna Centro, Facultad de Medicina, Universidad de Chile, Santiago, Chile

**Keywords:** COVID-19, humoral response, neutralization, organ transplantation, vaccine

## Abstract

**Introduction:**

Booster doses of SARS-CoV-2 vaccines improve seroconversion rates in solid organ transplant recipients (SOTRs) but the impact of homologous and heterologous booster doses in neutralizing antibody (NAb) titers and their ability to interfere with the variant of concern Omicron are not well studied.

**Methods:**

We designed a prospective, open-label, observational clinical cohort study. 45 participants received two doses of BNT162b2 or CoronaVac (21-day or 28-day intervals, respectively) followed by a first and second booster with BNT162b2 (5-month apart each) and we analyzed the neutralizing antibody titers against SARSCoV-2 D614G (B.1 lineage) and Omicron (BA.1 lineage).

**Results:**

Our results show that SOTRs receiving an initial two-dose scheme of CoronaVac or BNT162b2 generate lower NAbs titers against the ancestral variant of SARS-CoV-2 when compared with healthy controls. Although these NAb titers were further decreased against the SARS-CoV-2 Omicron, a single BNT162b2 booster in both groups was sufficient to increase NAb titers against the variant of concern. More importantly, this effect was only observed in those participants responding to the first two shots but not in those not responding to the initial vaccination scheme.

**Discussion:**

The data provided here demonstrate the importance of monitoring antibody responses in immunocompromised subjects when planning booster vaccination programs in this risk group.

## Introduction

Solid organ transplant recipients (SOTRs) are at increased risk for SARS-CoV-2 infection and remain at elevated mortality risk until the COVID-19 pandemic can be controlled (1). Different studies have shown low seroconversion rates of SOTRs that increased with one or two homologous booster doses of mRNA vaccines (2–6). However, studies analyzing and comparing neutralizing antibody (NAbs) titers elicited by different vaccine platforms or the impact of homologous versus heterologous boosters to neutralize the emerging variants of concern (VoCs), such as the Omicron variants in this group of severely immunosuppressed patients are limited (7–9).

To evaluate whether a fourth dose of the COVID-19 vaccine improves the neutralizing capacity in serum of SOTRs, we analyzed NAbs titers three months after an initial two-dose scheme of CoronaVac or BNT162b2; one month after 1^st^ booster and 16 days after 2^nd^ booster of BNT162b2 vaccine, in accordance with the booster vaccine policy implemented by the Chilean National Immunization Program (PNI), using an HIV-1-based SARS-CoV-2 pseudotype expressing the spike protein of the Omicron (BA.1 lineage) or SARS-CoV-2 D614G (B.1 lineage) (10–12).

## Materials and methods

### Study cohort

Healthcare workers without previously diagnosed SARS-CoV-2 infection and without the use of immunosuppressive drugs for any diagnostic from Clínica Santa María; Santiago, Chile and patients belonging to the transplant unit of the Clínica Santa Maria, Santiago, Chile were invited to participate in this study. Forty-five solid organ transplant recipients, 42.2% (19) women, with a mean age of 52 years (IQR 37-59) at the start of vaccination, were recruited. The transplanted organs were distributed as follows: 12 pulmonary (6 monopulmonary), 9 liver, 1 heart, 12 kidney and 11 kidney-pancreas transplants. More detailed information about the characteristics of the transplanted patients is shown in **Table 1**. Volunteers received the two-dose scheme of BNT162b2 (Pfizer-BioNTech) or CoronaVac (Sinovac Biotech), each dose being administered 21 or 28 days apart, respectively, according to the Chilean National Immunization Program (PNI). The participants subsequently received a first booster dose at day 148 (IQR 146-154) after the second initial dose and a second booster dose 153 (IQR 152-161) days after the first booster. All participants received both booster doses with BNT162b2 according to the regulations of the Ministry of Health. All participants were asked about their previous diagnosis of COVID-19 prior to every sample collection. If they had a record of a positive PCR, they were excluded from the analysis. The BNT162b2-vaccinated participants who tested positive for anti-N antibodies were discarded from the analysis.

**Table 1:** Characteristics of study subjects.

	Healthy volunteers	Solid organ transplant patients		
	sample after first 2 doses	sample after first 2 doses	sample after 1st booster	sample after 2° booster
**n**	50	45	19	20
**Female N (%)**	36 (72%)	19 (42.2%)	9 (47%)	8 (40%)
**Age (IQR)**	39.5 (30-51)	52 (37-59)	49 (37-60)	53 (39-64)
Months between transplant and start of vaccination (median -IQR))		24 (6.7 -47.9)	23.7 (6.5 - 48.0)	24 (6.9-48-4)
**Days between vaccine dose and sampling (IQR)**	99 (97-112)	90 (85-104)	36 (33-37)	16 (11-23)
				
**Solid organ transplant (n)**				
**Lung (mono or bi)**		12	5	8
**Liver**		9	4	3
**Heart**		1	0	0
**Kidney-pancreas**		11	6	6
**Kidney**		11	4	3
				
**Type of Immunosuppression (n)**				
**Steroids**		39	18	19
**Calcineurin inhibitors**		44	19	20
**Purine metabolism inh**		36	16	18
**m-TOR**		6	2	1

All participants signed informed consent before any study procedure was undertaken and protocols were approved by the respective Ethics Committee at Clínica Santa María (No. 132604-21) and Facultad de Medicina at Universidad de Chile (No. 0361-2021). Serum samples were collected between June 2021 and February 2022.

### Production of an HIV-1-based SARS-CoV-2-Spike pseudotyped virus

Pseudotyped viruses carrying SARS-CoV-2 Spike variants D614G (B.1 lineage) and Omicron (BA.1 lineage)) were produced as described in (11, 12). Briefly, HEK-293T cells were co-transfected with the HIV-1 proviral vector pNL4.3-ΔEnv-Luciferase and the corresponding pCDNA-SARS-CoV-2 Spike coding vectors using PEI. Spike codifying vectors were purchased from GenScript and designed to lack the last 19 amino acids of the C-terminal end (SΔ19) known to avoid retention at the endoplasmic reticulum. At 48 hours post-transfection, pseudotypes were recovered from the supernatant, cleared by centrifugation at 850g for 5 minutes at room temperature, diluted in 50% fetal bovine serum (Sigma-Aldrich), aliquoted and stored at -80°C until use. Pseudoviruses were quantified by HIV-1 Gag p24 Quantikine ELISA Kit (R&D Systems) following manufacturer’s instructions.

### Pseudotyped virus neutralization assay

Serum samples inactivated for 30 minutes at 56°C were 3-fold serially diluted (from 1:5 to 1:10935) in supplemented DMEM with 10% FBS. Samples were incubated with 3 ng of p24 HIV-1-based SARS-CoV-2 variant pseudotyped virus D614G (B.1 lineage) or Omicron (BA.1 lineage)) during 1 h at 37°C, and 1 × 10^4^ HEK-ACE2 cells were added to each well. HEK293T cells incubated with the pseudotyped virus were used as a negative control. Cells were lysed 48 h later, and firefly luciferase activity was measured using the Luciferase Assay Reagent (Promega) in a Glomax 96 Microplate luminometer (Promega). Relative luminescence units (RLUs) of HEK293T cells transduced with the corresponding pseudovirus were averaged and considered as 100% neutralization while RLUs measured at the highest dilution of each sample were established as 0% neutralization. Thus, the percentage of neutralization of each one of the eight dilutions of a sample was calculated as the complement of the division between the corresponding RLUs and the RLUs obtained at the higher dilution after subtracting the background (HEK293T + pseudovirus). This calculation was done independently for each technical replica and for each spike variant. Relative pseudotyped virus neutralization titer 50 (pVNT_50_) is defined as the dilution of the sample yielding a 50% diminution of firefly luciferase activity compared to the negative (HEK293T without pseudovirus) and positive controls (highest dilution of the sample). The pVNT_50_ was calculated in GraphPad Prism v9.1.2 (La Jolla, California, USA) by modeling a four-parameter non-linear regression with variable slope constraining top values to 100 and bottom values to 0. Samples showing a pVNT_50_ lower than the first dilution (1:10 for CoronaVac, 1:10 for BNT162b2) were considered as 10.

### Anti-Spike RBD antibodies determination

Quantification of anti-Spike RBD and anti-N antibodies was performed as described in (13) by using the Electrochemiluminescent immunoassay (ECLIA) (Cobas, Roche). Values are reported as the analyte concentration of each sample in U/mL. Detection ranges for anti-Spike RBD were 0.4 to 2500 U/mL, where a detection <0.8 U/mL was interpreted as negative and ≥0.80 U/mL was interpreted as positive for anti-Spike RBD antibodies. Detection of anti-N antibodies with a cut-off index ≥ 1.0 was considered as positive and <1 negative (Roche Diagnostics GmbH. Elecsys Anti-SARS-CoV-2 assay method sheet. 2021-03; version 4.0). Analysis of IgG and IgM antibodies anti-Spike and anti-N was evaluated by using the OnSite COVID-19 IgG/IgM Rapid Test Kit (CTK, Biotech) using 10 µL of serum samples following manufacturer’s instructions.

### Statistical analyses

Statistical analyses were performed using GraphPad Prism software v9.1.2 (La Jolla, California, USA). Multiple group comparisons for serum neutralization titers against a set of samples and the two SARS-CoV-2 pseudotyped viruses were applied using Kruskal-Wallis test with false discovery rate (FDR) method, and multiple testing correction was performed for each comparison using Benjamini-Hochberg (BH) procedure at a 5% FDR threshold. When indicated, factor change was calculated as the difference of geometric mean titer in the pVNT_50_ or total anti-Spike IgG levels. The degree of correlation between neutralizing and total IgG antibodies from different groups was evaluated by computing the Spearman’s ρ for every XY pair of values (13). A p-value ≤0.05 was considered statistically significant.

## Results

**Figure 1:**
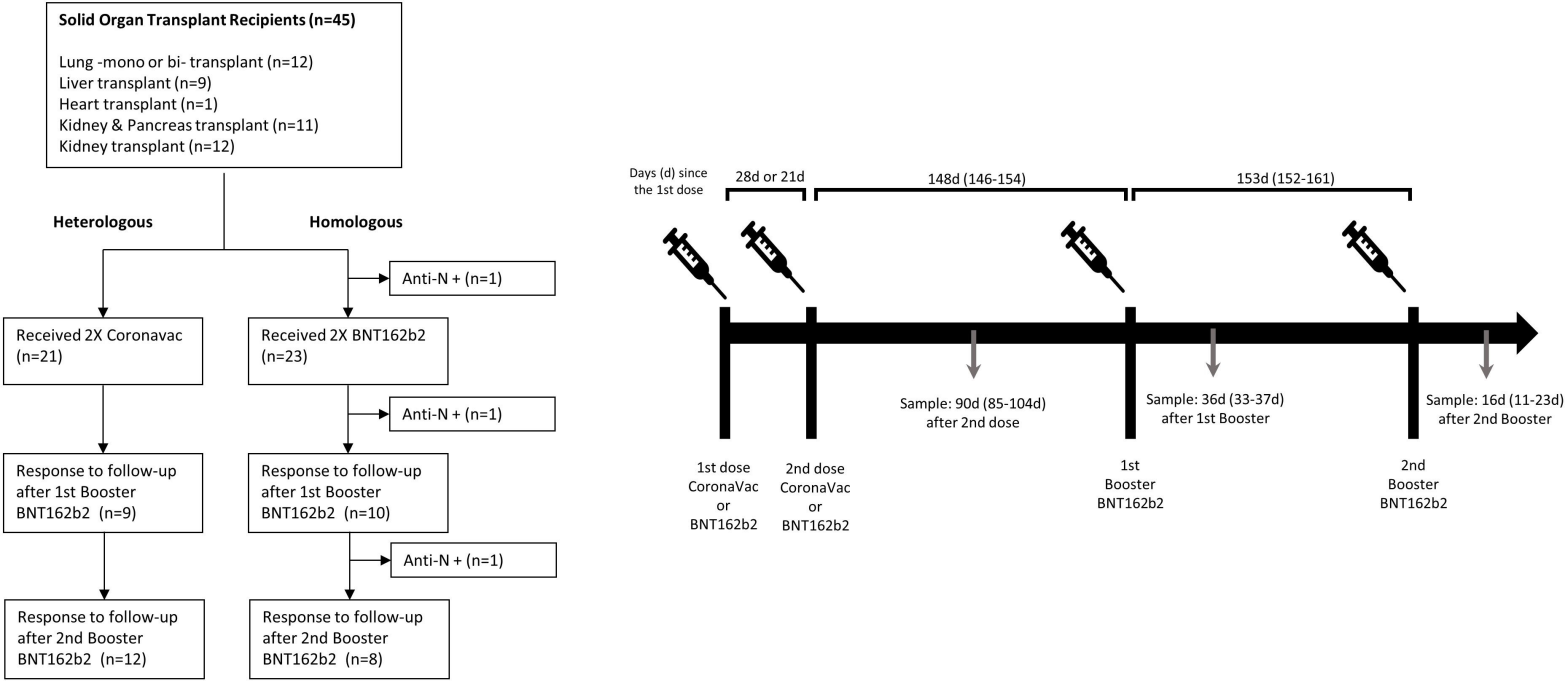
Study flow-chart and protocol of the observational clinical cohort study.

In this work, we determined NAbs titers in a cohort of SOTRs inoculated with the two-dose regimen of the mRNA vaccine BNT162b2 or the inactivated virus vaccine CoronaVac and receiving two boosters of BNT162b2 five months apart (**Figure 1**). Serum samples from SOT recipients (N=45, **Table 1**) and healthy healthcare workers (N=50, **Table 1**) were used to determine the neutralizing antibody (NAb) titers measured as pseudotyped virus neutralization titer 50 (pVNT_50_) as we have previously reported (10–12). Additionally, total IgG/IgM anti-Spike and anti-N antibodies were evaluated by a lateral flow rapid test and quantified by ELISA. Details regarding cohort demographics, methods and statistical analyses can be found in **Table 1** and **Supplementary Table 1**.

The vaccination process in SOTRs began at a median of 24.7 months (IQR 7.6 - 48) after the transplant. All the patients were on immunosuppression (described in **Table 1**). Only individuals without a clinical history of SARS-CoV-2 infection and without a history of positive PCR testing were analyzed. The participants received an initial vaccination schedule with two doses of the BNT162b2 vaccine or two doses of CoronaVac administered 21 or 28 days apart, respectively, then they all received the first booster with BNT162b2 at day 148 (IQR 146-154) after the second initial dose and a second booster 153 (IQR 152-161) days later (**Figure 1**). The initial vaccine scheme utilized in each patient was determined according to the vaccine that was available in the vaccination center when the immunization campaign began. Only a statistically significant difference in age was identified between the groups that received BNT162b2 versus CoronaVac at the initial scheme, with a median of 42 years for BNT162b2 and 57 years for CoronaVac (p-value = 0.0004). There was no difference in distribution based on sex, transplanted organ type, or immunosuppressive treatment.

In a previous report, we showed that neutralization levels of antibodies elicited by CoronaVac and BNT162b2 against the SARS-CoV-2 Wuhan-Hu-1 Spike are not affected by the D614G mutation that originated the B.1 lineage (11). Thus, we decided to conduct our analysis using Spike D614G- and Omicron-pseudotyped viruses.

**Figure 2:**
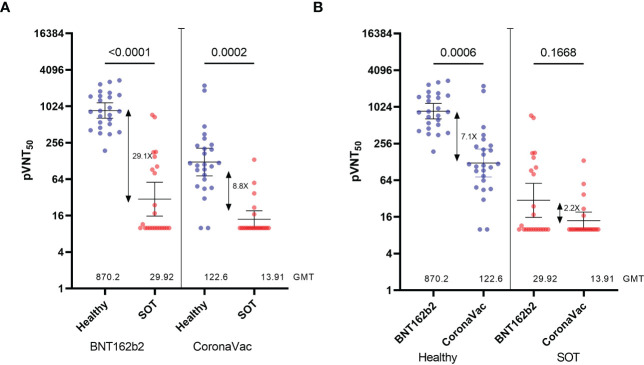
Neutralization titers of serum from Healthy and SOTRs 90 days after the two-dose BNT162b2 and CoronaVac vaccines. 50% pseudovirus neutralization titers (pVNT_50_) of 50 healthy recipients (blue) of the CoronaVac (n=25) and BNT162b2 (n=25) vaccines and 44 SOTRs (red) of the CoronaVac (n=21) and BNT162b2 (n=23) against pseudotypes **(A, B)** ancestral reference strain (D614G). Statistical significance of the difference between the neutralization was calculated by the two-tailed Kruskal–Wallis test after adjustment for the false discovery rate. Two-tailed *P* values are reported. Geometric mean titers (GMTs) and 95% CIs are indicated. Factor changes are shown in brackets as the difference of the geometric mean titer in the pVNT_50_. Graphs Y axes are presented in logarithmic (log_2_) scale.

First, we compared NAb titers induced by the two-dose scheme of BNT162b2 or CoronaVac in SOTRs and healthy volunteers against the D614G pseudovirus at 90 days after the second dose (**Figure 2A**). Consistent with a low seroconversion rate (BNT162b2 = 50%, CoronaVac=28.6%), NAb titers measured as the geometric mean of the pVNT_50_ were 29.1-fold and 8.8-fold lower for SOT recipients receiving BNT162b2 or CoronaVac, respectively, when compared with healthy controls whose seroconversion rate was 100%. Moreover, BNT162b2 elicited NAb titers that were 2.2-fold and 7.1-fold higher for SOT recipients and healthy controls, respectively, when compared with CoronaVac (**Figure 2B**). In the same line, anti-Spike RBD antibodies were 309.7-fold and 82.6-fold lower for BNT162b2- and CoronaVac-vaccinated SOTRs compared with healthy controls, respectively. Besides, as observed in the analysis of NAbs titers, anti-Spike RBD antibodies from healthy and SOT recipients vaccinated with BNT162b2 were 17.2-fold and 4.6-fold higher compared to CoronaVac (**Supplementary Figures 1A, B**). Interestingly, there was a strong correlation between NAbs measured with the HIV-1-based SARS-CoV-2-Spike pseudotyped virus and anti-Spike RBD antibodies from healthy- and SOTRs-BNT162b2 vaccinated groups (r=0.75 and r=0.7909, respectively) (**Supplementary Figure 1C**). However, the correlation was moderate in SOTRs receiving CoronaVac compared to the healthy control group (r=0.5633 versus r=0.7328), which further reinforces our conclusion that two doses of BNT162b2 elicit higher NAbs titers than CoronaVac in SOT patients (**Supplementary Figure 1C**).

Importantly, we assessed the seropositivity of the samples by a lateral flow rapid test kit to detect IgG anti-SARS-CoV-2 Spike antibodies (14). Globally (SOTRs + healthy), subjects with pVNT_50_ values near the detection limit (log(pVNT_50_<2.5)) were tested negative regardless initial vaccination scheme (CoronaVac or BNT162b2), whereas subjects with a log(pVNT_50_)>5 were positive for anti-SARS-CoV-2 antibodies and predominantly BNT162b2-vaccinated (**Supplementary Figure 2A**). The analysis of IgG positivity by group (SOTRs vs healthy) showed that healthy subjects with low NAbs titers elicited by CoronaVac (log(pVNT_50_<5) were associated to a higher occurrence of negative results, whilst the mRNA vaccine induced high NAbs titers that were detected as positive by the lateral flow kit (**Supplementary Figure 2B**). Consistent with a low seroconversion rate, CoronaVac- and BNT162b2-SOTRs were mainly IgG negative with pVNT_50_ values near the detection limit and presented a low occurrence of IgG positive results as well as NAbs titers that do not reach log(pVNT_50_) values higher than 5 (**Supplementary Figure 2C**).

**Figure 3:**
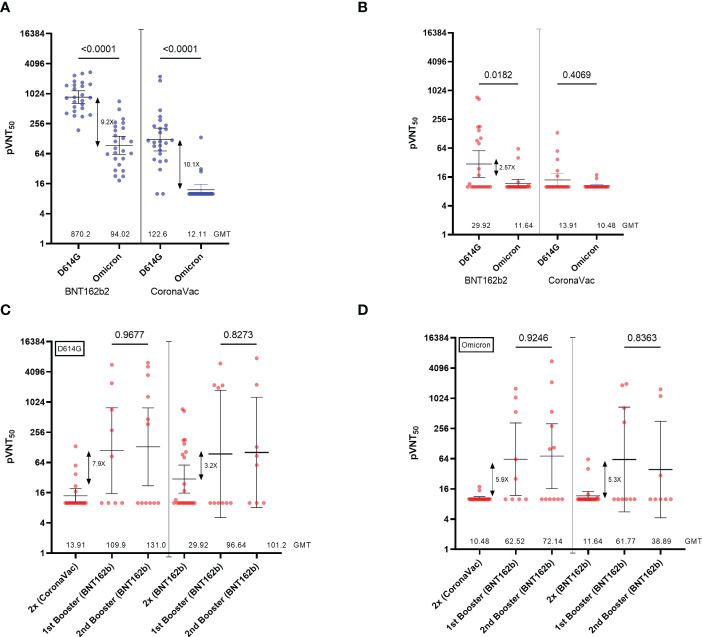
Neutralization titers of serum from SOTRs across the homologous and heterologous boosters. **(A)** 50% pseudovirus neutralization titers (pVNT_50_) of 50 healthy recipients (blue) of the CoronaVac (n=25) and BNT162b2 (n=25) vaccines and **(B)** 44 SOT recipients (red) of the CoronaVac (n=21) and BNT162b2 (n=23) against pseudotypes ancestral reference strain (D614G) or Omicron (BA.1). **(C, D)** 50% pseudovirus neutralization titers (pVNT_50_) of 44 SOT recipients of the CoronaVac (n=21; 1^st^ booster n=9, 2^nd^ booster n=12)) and BNT162b2 (n=23; 1^st^ booster n=10; 2^nd^ booster n=8)) against pseudotypes **(C)** ancestral reference strain (D614G) or **(D)** Omicron (BA.1). Geometric mean titers (GMTs) and 95% CIs are indicated. Factor changes are shown in brackets as the difference of the geometric mean titer in the pVNT_50_. Statistical significance of the difference between the neutralization was calculated by the two-tailed Kruskal–Wallis test after adjustment for the false discovery rate. Two-tailed *P* values are reported. Graphs Y axes are presented in logarithmic (log_2_) scale.

We then looked at the neutralizing ability of antibodies against the SARS-CoV-2 variant of concern Omicron (**Figure 3A**). As expected, we observed a 9.2-fold and 10.1-fold decrease in the NAb titers in the healthy group inoculated with BNT162b2 and CoronaVac, respectively, compared to NAb titers against the reference strain D614G (**Figure 3A**). While NAb titers from the SOT recipients group inoculated with CoronaVac were near the limit of detection (pVNT_50_ <10), the low but detectable NAb titers against the D614G pseudovirus in the group of SOT recipients inoculated with BNT162b2 were decreased by 2.57-fold against Omicron (**Figure 3B**). These data show that a two-dose regimen of SARS-CoV-2 vaccines BNT162b2 and CoronaVac in SOT recipients elicits very low levels of NAbs titers, which are higher in those inoculated with the mRNA vaccine. However, these NAbs titers are not sufficient to neutralize the Omicron variant.

We then analyzed the impact of one and two BNT162b2 booster doses inoculated with a 5-month interval in those SOT recipients who do not drop out of the study (CoronaVac (n=21; 1^st^ booster n=9, 2^nd^ booster n=12) and BNT162b (n=23; 1^st^ booster n=10; 2^nd^ booster n=8)). We observed that while a single heterologous booster dose (2x CoronaVac + 1x BNT162b2) induced a 7.9-fold increase in NAb titers, participants who received a homologous booster (2x BNT162b2 + 1x BNT162b2) increased their NAbs titers against the D614G pseudovirus by 3.2-fold (**Figure 3C**). Different from what has been previously reported for healthy patients (15–18), we observed that a second booster with BNT162b2 (fourth dose) in both settings did not have major effects on NAb titers over the first booster in SOTRs (**Figure 3C**). Consistent with the analysis of NAbs, total anti-Spike IgG antibodies increased by 13.5-fold and 7.9-fold after a heterologous and homologous booster, respectively (**Supplementary Figure 3**), while no significant differences were observed after the second booster. Of note, we observed that 50% of the patients included in the follow-up did not respond to any of the boosters regardless of the initial vaccination scheme (pVNT_50_ <10). Indeed, only 1 of the 6 (16.6%) SOTRs that did not seroconvert after the initial two-dose scheme turned positive for anti-Spike RBD antibodies after the first BNT162b2 booster. Moreover, 2 of the 7 (28.6%) CoronaVac- and 3 of the 6 (50%) BNT162b2-initially vaccinated SOTRs seroconverted solely after the second booster. In this manner, the cumulative percentage of seropositive SOTRs after the second booster dose was higher when receiving a homologous (75%) versus a heterologous (53%) vaccination scheme.

Finally, we evaluated whether NAbs induced by one or two booster doses were able to neutralize the Omicron variant. Interestingly, NAbs elicited by a heterologous booster in SOTRs neutralize the Omicron variant in a 5.9-fold increase (**Figure 3D**). Similar results were obtained in those SOTRs that generated NAbs followed by a homologous booster showing a 5.3-fold increase. We also observed that a second booster with BNT162b2 (fourth dose) in both settings did not have major effects on NAb titers over the first booster in SOTRs against the Omicron variant (**Figure 3D**).

## Discussion

In the present study, we show that SOTRs have weak neutralizing antibody responses against the SARS-CoV-2 B.1 and Omicron BA.1 variants even after two boosters with the BNT162b2 mRNA vaccine. Importantly, those SOTRs not responding to the first vaccination scheme do not show an increase in their NAb titers upon one and two boosters (19, 20). However, we detected an important increase in cumulative seroconversion rates, especially after the second booster under a homologous scheme. Limitations of this study include a low number of volunteers, that some volunteers failed to respond at follow-up, and the lack of data on B and T cell responses, which may provide antibody-independent protection.

Recently, a meta-analysis showed that booster vaccination enhances the immunogenicity of COVID-19 vaccines in SOTRs, however, a significant share of the recipients still has not built a detectable humoral immune response after the 3rd dose (21). Our results are also in line with studies in other groups at high risk of developing COVID-19 such as haemodialysis (HD) patients. Similar to SOTRs, HD patients present lower antibody titers and seroconversion rates after a two-dose BNT162b2 vaccination scheme compared to healthy controls, which can be significantly increased after a third BNT162b2 dose (22, 23). This confirms the urgent necessity of maintaining a booster dose in SOTRs at each 5-month interval and provides evidence that the use of the mRNA-based vaccines as boosters are sufficient to increase NAb titers able to neutralize the SARS-CoV-2 variant of concern Omicron. Interestingly, we showed that serum reactivity against RBD (ECLIA) as well as IgG positivity (OnSite) are associated to NAb titers predominantly when pVNT_50_ values are medium-to-high according to our assay’s range. Thus, the data provided here highlight the importance of monitoring antibody responses in immunocompromised subjects, which according to our results should be considered when planning vaccination programs in these risk groups. While different strategies such as the use of monoclonal antibodies for early treatment or prophylaxis, convalescent plasma, drugs such as nirmatrelvir/ritonavir, molnupiravir and remdesivir, anti-inflammatory therapy, and virus specific T-cell therapy are being evaluated (24). we urgently need to find alternative approaches for this specific set of patients.

## Data availability statement

The original contributions presented in the study are included in the article/**Supplementary Material**. Further inquiries can be directed to the corresponding authors.

## Ethics statement

All participants signed informed consent before any study procedure was undertaken and protocols were approved by the respective Ethics Committee at Clínica Santa María (No. 132604-21) and Facultad de Medicina at Universidad de Chile (No. 0361-2021). The patients/participants provided their written informed consent to participate in this study.

## Authors contributions

AG-A, CPC, FV-E, and RS-R designed the study. MAl and CPC provided clinical samples. AG-A, VS-A, LA-P and MAc performed neutralization assays. SB and AK provided reagents and plasmids. AG-A, DS and FV-E performed the statistical analysis. AG-A, FV-E, CPC, and RS-R wrote the manuscript. RS-R, FV-E and CPC acquired funding. All authors contributed to the article and approved the submitted version.

## Supplementary material

Supplementary Figure 1 Anti-Spike RBD antibodies of serum from Healthy and SOTRs 90 days after the two-dose BNT162b2 and CoronaVac vaccines. **(A, B)** Results of serological assay measuring serum reactivity to RBD expressed as U/ml (SARS-CoV-2 positive ≥0.80 U/ml) (Elecsys^®^, Roche Diagnostic GmbH)) from 41 healthy recipients (blue) of the CoronaVac (n=17) and BNT162b2 (n=24) vaccines and 44 SOTRs (red) of the CoronaVac (n=21) and BNT162b2 (n=23). Differences in the geometric means titers of anti-Spike RBD between CoronaVac and BNT162b2 vaccine are shown. Statistical analyses were performed with the two-tailed Kruskal–Wallis test after adjustment for the false discovery rate. **(C)** Correlation between NAbs measured as the pVNT_50_ and anti-Spike RBD antibodies in U/mL for each group described in **(A)**. Spearman r and two-tailed p-value are reported in the legend. The dashed line shows the detection limit (0.8 U/mL) of the technique. Values are presented in log10 scale.

Supplementary Figure 2 Relation between IgG positivity and NAb titers against the ancestral reference strain (D614G) of serum from Healthy and SOTRs individuals. **(A)** Frequency of IgG positive and negative results evaluated by lateral flow rapid test kit as a function of the natural logarithm of the pVNT_50_ value from CoronaVac- or BNT162b2- vaccinated healthy and SOTRs individuals included in this study (n=88). Distribution of IgG positive and negative results occurrence from CoronaVac- or BNT162b2- vaccinated healthy (n=40) **(B)** and SOTRs (n=44) **(C)** compared to NAb titers (log(pVNT_50_)) against the ancestral reference strain (D614G).

Supplementary Figure 3 Anti-Spike RBD antibodies of serum from SOT individuals across the homologous and heterologous boosters. Results of serological assay measuring serum reactivity to RBD expressed as U/ml (SARS-CoV-2 positive ≥0.80 U/ml) (Elecsys^®^, Roche Diagnostic GmbH)) from 44 SOTRs of the CoronaVac (n=21; 1st booster n=9, 2nd booster n=12)) and BNT162b2 (n=23; 1st booster n=10; 2nd booster n=8)). Geometric mean titers (GMTs) and 95% CIs are indicated. Factor changes are shown in brackets as the difference of the geometric mean titer. Statistical significance of the difference between the anti-Spike RBD titers was calculated by the two-tailed Kruskal–Wallis test after adjustment for the false discovery rate. Two-tailed P values are reported. Graphs Y axes are presented in logarithmic (log2) scale. The dashed line shows the detection limit (0.8 U/mL) of the technique.

Supplementary Table 1 Detailed information about the characteristics of the transplanted patients (SOTRs) and healthy controls (columns A-G). Results of neutralization assays (pVNT_50_, confidence intervals and R^2^), ELISA anti-Spike and anti-N as well as OnSite rapid test of serums at 90 days after the initial vaccination scheme (columns I-S), after the first booster (columns U-AF) and after the second booster (AH-AS) of each sample are shown. Excluded samples tested positive for anti-N antibodies are highlighted in yellow (“NA”, non-applicable; “–”, no data; ND, non-determined).
