# Supplementary figures and images for: Impact of homologous and heterologous boosters in neutralizing antibodies titers against SARS-CoV-2 Omicron in solid-organ transplant recipients
Source: Front Immunol. 2023 Mar 13;14:1135478. doi: 10.3389/fimmu.2023.1135478 (PMC10044136; doi:10.3389/fimmu.2023.1135478)

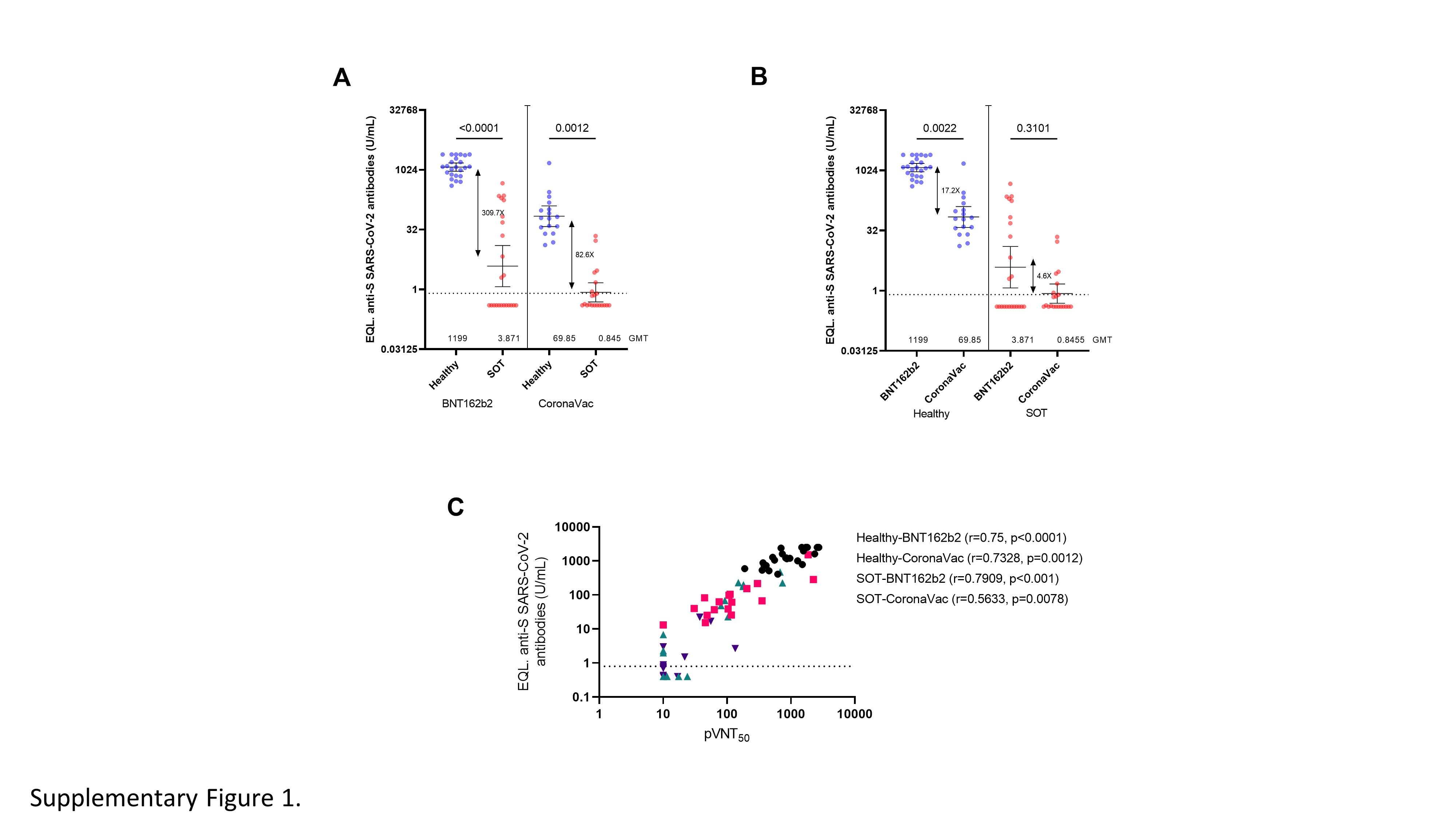

Supplement: Supplementary Figure 1 — Anti-Spike RBD antibodies of serum from Healthy and SOTRs 90 days after the two-dose BNT162b2 and CoronaVac vaccines. (A, B) Results of serological assay measuring serum reactivity to RBD expressed as U/ml (SARS-CoV-2 positive ≥0.80 U/ml) (Elecsys®, Roche Diagnostic GmbH)) from 41 healthy recipients (blue) of the CoronaVac (n=17) and BNT162b2 (n=24) vaccines and 44 SOTRs (red) of the CoronaVac (n=21) and BNT162b2 (n=23). Differences in the geometric means titers of anti-Spike RBD between CoronaVac and BNT162b2 vaccine are shown. Statistical analyses were performed with the two-tailed Kruskal–Wallis test after adjustment for the false discovery rate. (C) Correlation between NAbs measured as the pVNT50 and anti-Spike RBD antibodies in U/mL for each group described in (A). Spearman r and two-tailed p-value are reported in the legend. The dashed line shows the detection limit (0.8 U/mL) of the technique. Values are presented in log10 scale. [file Image_1.jpeg]

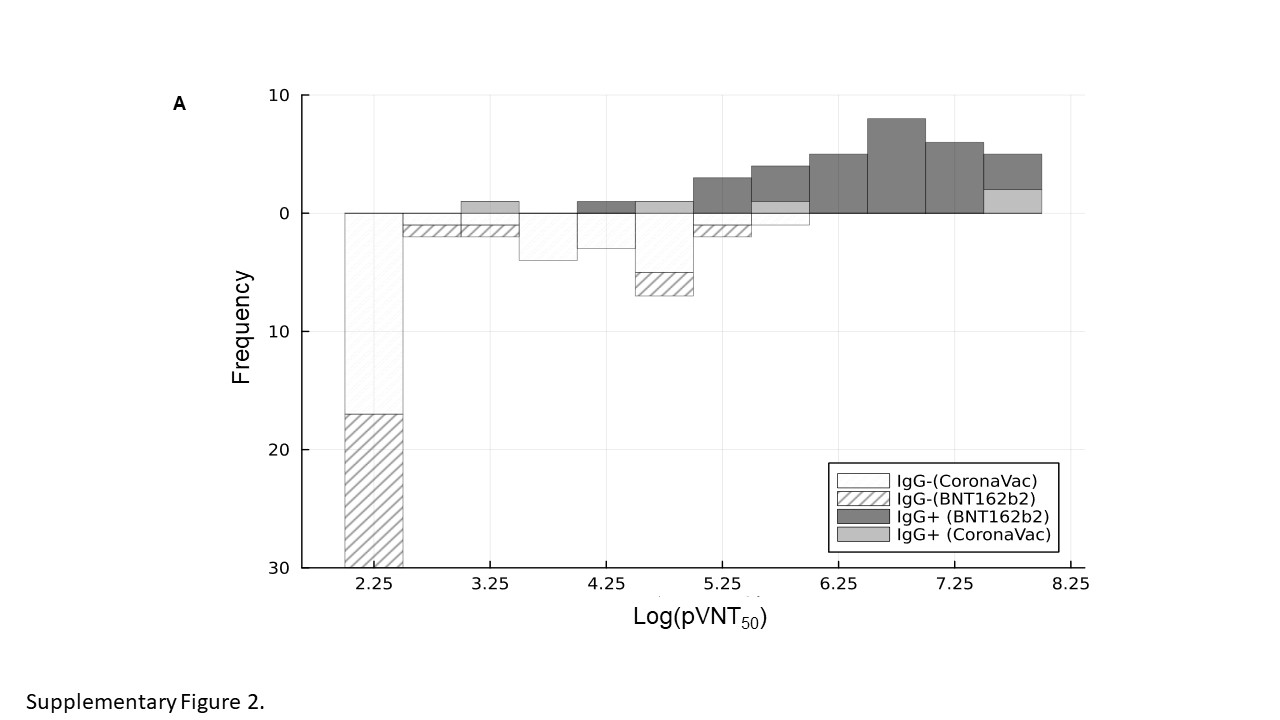

Supplement: Supplementary Figure 2 — Relation between IgG positivity and NAb titers against the ancestral reference strain (D614G) of serum from Healthy and SOTRs individuals. (A) Frequency of IgG positive and negative results evaluated by lateral flow rapid test kit as a function of the natural logarithm of the pVNT50 value from CoronaVac- or BNT162b2- vaccinated healthy and SOTRs individuals included in this study (n=88). Distribution of IgG positive and negative results occurrence from CoronaVac- or BNT162b2- vaccinated healthy (n=40) (B) and SOTRs (n=44) (C) compared to NAb titers (log(pVNT50)) against the ancestral reference strain (D614G). [file Image_2.jpeg]

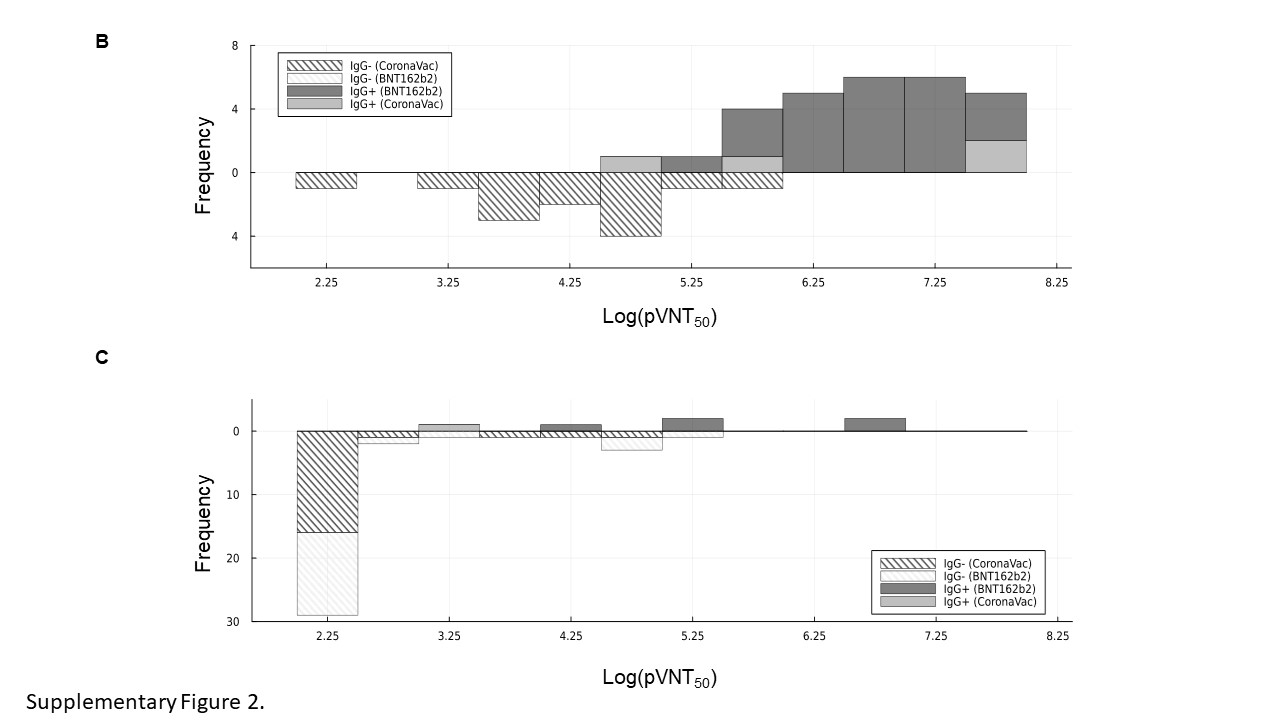

Supplement: Supplementary Figure 3 — Anti-Spike RBD antibodies of serum from SOT individuals across the homologous and heterologous boosters. Results of serological assay measuring serum reactivity to RBD expressed as U/ml (SARS-CoV-2 positive ≥0.80 U/ml) (Elecsys®, Roche Diagnostic GmbH)) from 44 SOTRs of the CoronaVac (n=21; 1st booster n=9, 2nd booster n=12)) and BNT162b2 (n=23; 1st booster n=10; 2nd booster n=8)). Geometric mean titers (GMTs) and 95% CIs are indicated. Factor changes are shown in brackets as the difference of the geometric mean titer. Statistical significance of the difference between the anti-Spike RBD titers was calculated by the two-tailed Kruskal–Wallis test after adjustment for the false discovery rate. Two-tailed P values are reported. Graphs Y axes are presented in logarithmic (log2) scale. The dashed line shows the detection limit (0.8 U/mL) of the technique. [file Image_3.jpeg]

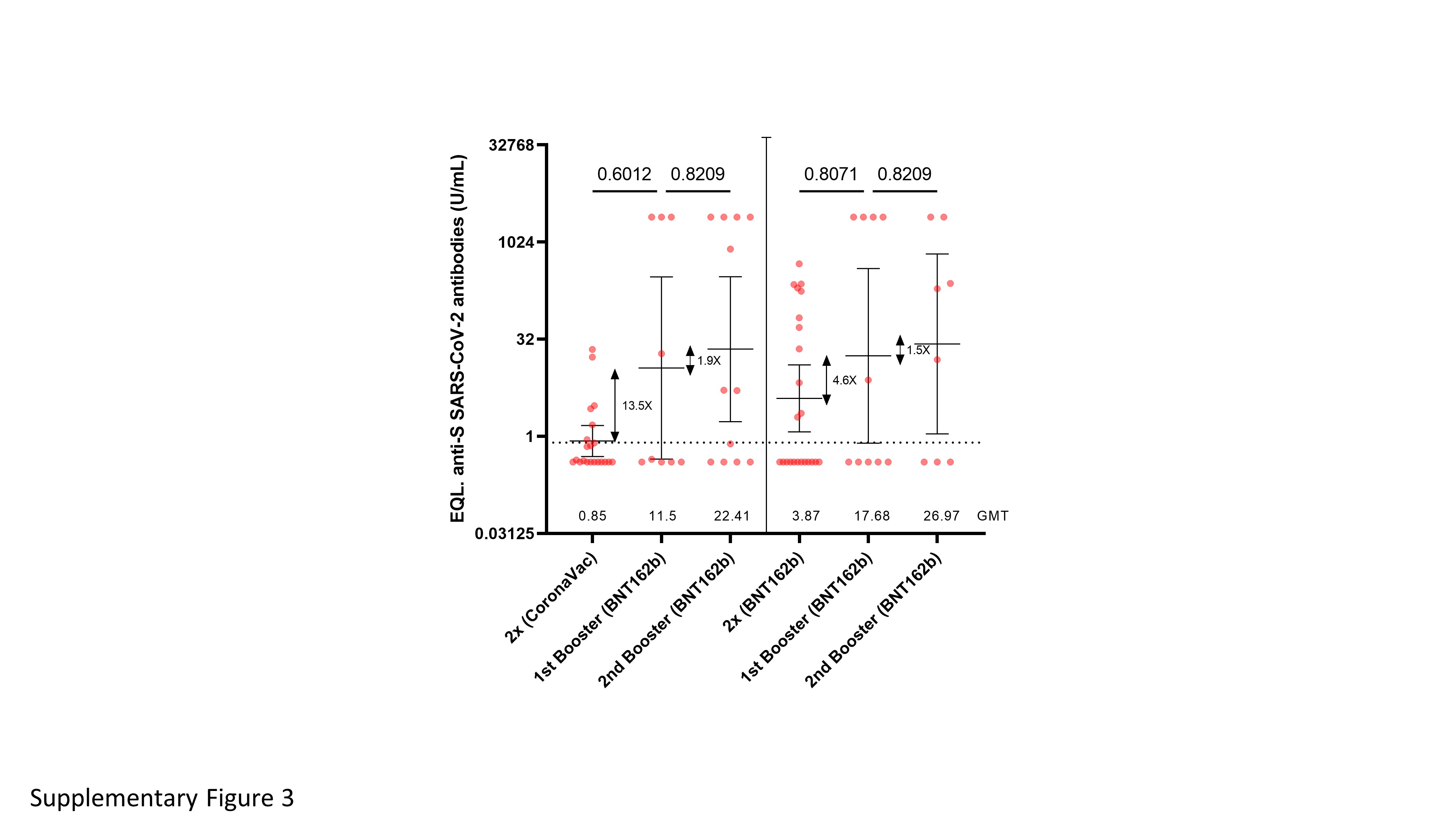

Supplement: Supplementary Table 1 — Detailed information about the characteristics of the transplanted patients (SOTRs) and healthy controls (columns A-G). Results of neutralization assays (pVNT50, confidence intervals and R2), ELISA anti-Spike and anti-N as well as OnSite rapid test of serums at 90 days after the initial vaccination scheme (columns I-S), after the first booster (columns U-AF) and after the second booster (AH-AS) of each sample are shown. Excluded samples tested positive for anti-N antibodies are highlighted in yellow (“NA”, non-applicable; “–”, no data; ND, non-determined). [file Image_4.jpeg]
